# DIFFERENTIAL PAIN SENSATION AND GUT MICROBIOME BETWEEN YOUNG AND OLDER ADULTS

**DOI:** 10.1093/geroni/igad104.2582

**Published:** 2023-12-21

**Authors:** Jie Chen, Zequan Wang, Angela Starkweather, Ming-Hui Chen, Hongyu Miao, Hyochol Ahn, Xiaomei Cong

**Affiliations:** Florida State University, Tallahassee, Florida, United States; University of Connecticut, Storrs, Connecticut, United States; University of Florida, Gainesville, Florida, United States; University of Connecticut, Storrs, Connecticut, United States; Florida State University, Tallahassee, Florida, United States; Florida State University, Tallahassee, Florida, United States; Yale University, New Haven, Connecticut, United States

## Abstract

This study aimed to investigate the differences in pain sensation and gut microbiota between young and older adults. Twenty-one healthy young adults (YAs) and sixteen healthy older adults (OAs) were recruited. Quantitative sensory testing data, and stool samples were collected. The 16S rRNA V4 gene region of stool samples were sequenced and processed by using the Mothur 1.42.3 pipeline. The mean ages of YAs and OAs were 20.14 (SD = 1.39) and 63.19 (SD = 8.00), respectively. Eleven of the YAs and 12 of the OAs were female, and 8 of the YAs and 3 of the OAs reported pain. OAs had significantly higher heat pain threshold (HPT), higher pressure pain threshold (PPT), lower cold detection threshold (CDT), and lower cold pain threshold (CPT) than YAs (all p < 0.05). OAs had significantly different a-diversity indices including higher species of observed (Sobs), Chao index and Shannon index, and lower coverage, and significantly different beta diversity indices (Bray-Curtis, Jaccard, and Theta YC) than YAs. The significantly abundant taxa in YAs included Bacteroidaceae, Veillonellaceae, and Alcaligenaceae. Coriobacteriaceae, Streptococcaceae, Lachnospiraceae, Erysipelotrichaceae, and Verrucomicrobiaceae were significantly enriched in OAs. The Spearman correlation analysis showed that the CDT, CPT, and HPT were significantly associated with gut microbiota Sobs, Chao index, Shannon index, and coverage (all p < 0.05). But the directions and coefficients of correlations varied. OAs had distinguished peripheral sensation and gut microbiota profiles than YAs. The changed peripheral sensation and gut microbiota profiles among OAs may be biomarkers of aging-related pain processing.

